# Cognitive behavioral therapy with interoceptive exposure and complementary video materials for irritable bowel syndrome (IBS): protocol for a multicenter randomized controlled trial in Japan

**DOI:** 10.1186/s13030-019-0155-2

**Published:** 2019-06-06

**Authors:** Hitomi Kawanishi, Atsushi Sekiguchi, Misako Funaba, Yasushi Fujii, Kazuhiro Yoshiuchi, Hiroe Kikuchi, Keisuke Kawai, Kazushi Maruo, Norio Sugawara, Kenji Hatano, Tomotaka Shoji, Tadahiro Yamazaki, Kenta Toda, Masafumi Murakami, Masayasu Shoji, Chisato Ohara, Yoshitoshi Tomita, Shin Fukudo, Tetsuya Ando

**Affiliations:** 1 Department of Behavioral Medicine, National Institute of Mental Health, National Center of Neurology and Psychiatry (NCNP), Kodaira, 187-8553 Japan; 2 Department of Psychology, Meisei University, Hino, Japan; 3 Graduate School of Medicine, The University of Tokyo, Tokyo, Japan; 4 Department of Psychosomatic Medicine, Center Hospital, National Center for Global Health and Medicine, Tokyo, Japan; 5 Department of Psychosomatic Medicine, Kohnodai Hospital, National Center for Global Health and Medicine, Ichikawa, Japan; 6 Translational Medical Center, National Center of Neurology and Psychiatry, Kodaira, Japan; 7 Department of Biostatistics, Faculty of Medicine, University of Tsukuba, Tsukuba, Japan; 8 Department of Behavioral Medicine, Graduate School of Medicine, Tohoku University, Sendai, Japan; 9 Department of Psychosomatic Medicine, Tohoku University Hospital, Sendai, Japan; 10 Department of Psychosomatic Medicine, National Center Hospital of Neurology and Psychiatry, Kodaira, Japan

**Keywords:** Irritable bowel syndrome, Cognitive behavioral therapy, Interoceptive exposure, Randomized controlled trial, Refractory IBS, Video materials, Home-based self-management, Multicenter

## Abstract

**Background:**

There is growing evidence of the treatment efficacy of cognitive behavioral therapy (CBT) for irritable bowel syndrome (IBS). CBT is recommended by several practice guidelines for patients with IBS if lifestyle advice or pharmacotherapy has been ineffective. Manual-based CBT using interoceptive exposure (IE), which focuses on the anxiety response to abdominal symptoms, has been reported to be more effective than other types of CBT. One flaw of CBT use in general practice is that it is time and effort consuming for therapists. Therefore, we developed a set of complementary video materials that include psycho-education and homework instructions for CBT patients, reducing time spent in face-to-face sessions while maintaining treatment effects. The purpose of this study is to examine the effects of CBT-IE with complementary video materials (CBT-IE-w/vid) in a multicenter randomized controlled trial (RCT).

**Methods:**

This study will be a multicenter, parallel-design RCT. Participants diagnosed with IBS according to the Rome IV diagnostic criteria will be randomized to either the treatment as usual (TAU) group or the CBT-IE-w/vid + TAU group. CBT-IE-w/vid consists of 10 sessions (approximately 30 min face-to-face therapy + viewing a video prior to each session). Patients in the CBT-IE-w/vid group will be instructed to pre- view 3- to 13-min videos at home prior to each face-to-face therapy visit at a hospital. The primary outcome is the severity of IBS symptoms. All participants will be assessed at baseline, mid-treatment, post-treatment, and follow-up (3 months after post assessment). The sample will include 60 participants in each group.

**Discussion:**

To our knowledge, this study will be the first RCT of manual-based CBT for IBS in Japan. By using psycho-educational video materials, the time and cost of therapy will be reduced. Manual based CBTs for IBS have not been widely adopted in Japan to date. If our CBT-IE-w/vid program is confirmed to be more effective than TAU, it will facilitate dissemination of cost-effective manual-based CBT in clinical settings.

**Trial registration:**

The trial was registered to the University Hospital Medical Information Network Clinical Trial Registry: UMIN, No. UMIN000030620 (Date of registration: December 28, 2017).

## Introduction

Irritable bowel syndrome (IBS) is a common chronic functional gastrointestinal disorder that affects about 14% [1] of the Japanese population. Symptoms of IBS such as abdominal pain and altered bowel habits (i.e., constipation and/or diarrhea) interfere with the patient’s quality of life (QOL) [2] and social functioning [3, 4]. IBS is associated with a significant increase in time off work [5] and increased health care use. Severity of abdominal pain/discomfort is a significant predictor of health care use for patients with IBS compared with non-IBS subjects [6]. Annual medical expenses paid for patients with IBS were reported to be 50% higher than those for population-controlled non-IBS patients [7].

Evidence for IBS treatment efficacy using cognitive behavioral therapy (CBT) has been growing [8–10]. Systematic reviews and meta-analysis have repeatedly confirmed CBT efficacy for IBS [11, 12]. Treatment guidelines issued by the National Institute of Clinical Excellence (NICE) in the United Kingdom recommend introducing CBT if lifestyle advise or pharmacotherapies have been ineffective and symptom duration has exceeded 12 months [13]. Specific psychotherapies including CBT are also recommended in the clinical guideline issued by the Japanese Society of Gastroenterology as the third step of IBS treatment that has been refractory to the first step (lifestyle advise and gut-targeted pharmacotherapies) and second step (psychopharmacological agents and brief psychotherapies) treatments [14]. However, CBT for IBS is available only in specific centers in Japan and evidence of its efficacy has not been established here. Therefore, RCT evidence of efficacy for CBT for patients with IBS is needed.

Among the several CBT programs reported for IBS, we adopted the interoceptive exposure-based CBT program (CBT-IE) for IBS developed by Craske et al. [15]. CBT-IE for IBS includes exposure to abdominal sensations in addition to psychoeducation, self-monitoring, cognitive restructuring, attention training, and in vivo exposure, which are often used in traditional CBT. Patients are exposed to self-induced abdominal sensations (e.g., by tightening abdominal muscles, or consuming avoided foods, etc.) to reduce anxiety in response to abdominal disturbance common in IBS.

Interoceptive exposure (IE) was originally developed for panic disorder [16], and its clinical applications have been expanded. IE is considered to weaken the fear response by enabling new learning that competes with the initial fearful associations [17]. It has been suggested that gastrointestinal symptom-specific anxiety (GSA) is important in perpetuating IBS. GSA has been shown to predict symptom severity and QOL in IBS. The effect on IBS symptoms and QOL of interventions using exposure that targets GSA has been supported by previous studies [18, 19], and its superiority over active treatment controls has been shown in some RCTs [15, 20].

Our single-arm feasibility study of CBT-IE for Japanese IBS patients [21] indicated significant reduction of IBS symptoms and remarkable improvement in IBS-specific QOL at post-intervention, 3-month, and 6-month follow-ups compared with the pre-intervention state. Although CBT-IE appears to be a promising treatment for refractory IBS, there are some difficulties in disseminating this intervention to clinical settings in Japan, including the limited number of CBT therapists and the highly time-consuming process of CBT [11].

To reduce the implementation costs, we developed a set of complementary video materials that cover psychoeducation and homework instructions regarding treatment so that patients can prepare at home for the subsequent in hospital, face-to-face therapy. The treatment procedures and contents are the same as with the original CBT-IE. Our additional feasibility study indicated that use of the video material with CBT-IE reduced the time spent in face-to-face therapy by about 40% on average, while treatment effectiveness remained constant. We calculated effect sizes (Hedges’ g) for changes in the Japanese version of the IBS Severity Index (IBS-SI-J) before and after the CBT-IE intervention. The effect size of our first feasibility study of face-to-face therapy alone [21] was g = − 1.02 (*N* = 20, session time: 66 ± 7 min), and the effect size of our second feasibility study using video material [22] was g = − 0.99 (*N* = 8, session time: 38 ± 7 min).

### Research objectives and hypotheses

An RCT will be done to examine the efficacy of CBT-IE with complementary video material (CBT-IE-w/vid) for Japanese patients with moderate to severe refractory IBS patients. The hypothesis is that participants allocated to the CBT-IE-w/vid + TAU group will have significantly reduced symptoms compared to the participants in the TAU alone group.

## Methods

This multicenter RCT will be conducted by a multidisciplinary team of medical doctors who specialize in psychosomatic medicine and functional gastrointestinal disorders, clinical psychotherapists, researchers, and statisticians.

### Design

A multicenter randomized controlled trial with two equal-sized parallel groups at five centers.

**Fig. 1:**
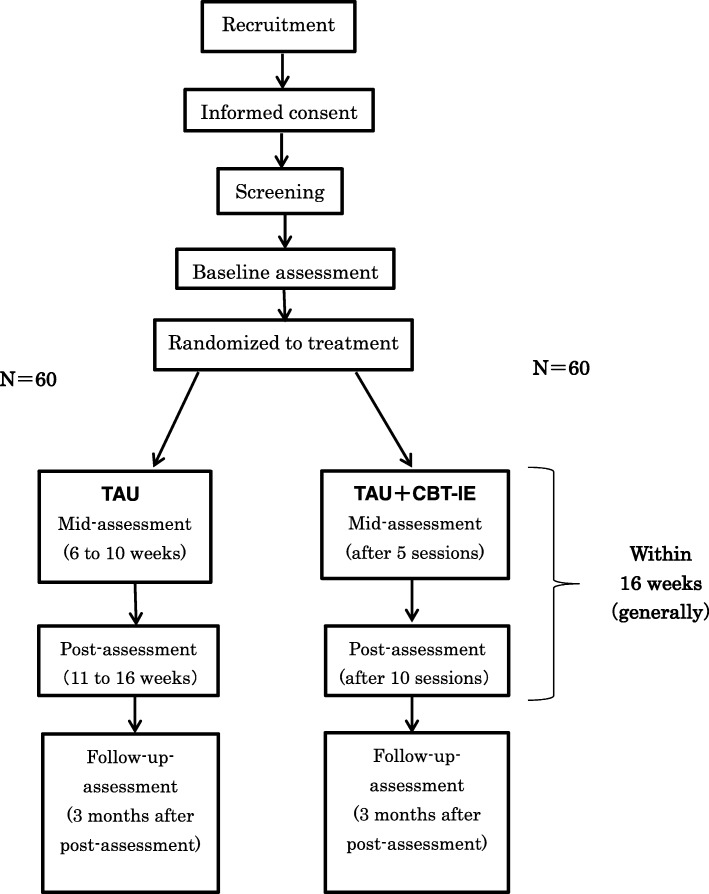
Flow of participants through the study

Potential participants will be screened for eligibility and will give written informed consent to participate. All participants will be assessed at baseline and then individually randomized into the CBT-IE-w/vid + TAU or TAU alone groups. Participant flow is depicted in Fig. 1.

All assessment measures, except for screening, are self-report questionnaires. Participants will complete baseline (pre-treatment), mid-treatment, post-treatment, and 3-month follow-up assessments. This protocol has been reviewed and approved by the Institutional Review Board of the National Center of Neurology and Psychiatry (accepted on November 29, 2017; A2017–067) and by the Ethics Committees of the four other collaborating facilities. The trial has been registered in the University Hospital Medical Information Network (UMIN) Clinical Trials Registry (URL: http://www.umin.ac.jp), No. UMIN000030620.

### Setting

Participants assigned to the CBT-IE-w/vid + TAU group will receive the standard IBS treatment from a medical doctor and face-to-face CBT-IE sessions with a therapist (a clinical psychotherapist or medical doctor who specializes in psychosomatic medicine) at a hospital. In addition, participants in the CBT-IE group will be instructed to watch the complementary video material and complete homework at home. Participants in the TAU alone group will receive the standard IBS treatment from a medical doctor at the hospital.

The intervention will be conducted at outpatient units of the five collaborating hospitals: National Center Hospital, National Center of Neurology and Psychiatry, Tohoku University Hospital, the University of Tokyo Hospital, Center Hospital of the National Center for Global Health and Medicine, and Kohnodai Hospital, National Center for Global Health and Medicine in Japan.

### Target population

Participants will be recruited from the five hospitals listed above and will include 120 outpatients aged 16 years or older with an IBS diagnosis according to the Rome IV diagnostic criteria and moderate to severe IBS symptoms based on IBS-SI-J scores.

### Recruitment

Recruitment will be conducted at each center by advertising and referrals from health care professionals. When a health care professional deems CBT-IE-w/vid feasible for the patient, the patient will be informed of this trial and referred to the trial team of each center.

### Screening

The initial screening will be conducted by medical doctors of the trial team of each center, all of whom specialize in psychosomatic medicine. After the patients give informed consent, eligibility will be evaluated according to the inclusion and exclusion criteria. Eligible participants will be provisionally registered, subjected to the baseline outcome assessment, and then definitively registered.

### Eligibility criteria

#### Inclusion criteria

To be eligible to participate in this study, a participant must meet all of the following criteria:

1. Aged 16 years or older with IBS that is refractory to standard pharmacotherapy
2. Diagnosed with IBS based on the Rome IV diagnostic criteria [23]
3. The severity of IBS is moderate or severe (IBS-SI-J score: 175 or over) [24, 25]
4. Able to understand the purpose and contents of this trial and give voluntary written informed consent

#### Exclusion criteria

A potential participant who meets any of the following criteria will be excluded from participation:

1. Organic disease suggested by the presence of warning symptoms
2. A history of concomitant inflammatory bowel disease, malignant tumor, or other bowel disease which could cause the current bowel symptoms
3. A comorbidity of major psychiatric disease, such as psychotic disorders, bipolar disorder, substance abuse-related disorders, eating disorders, obsessive and compulsive disorders, or PTSD. However, persons with anxiety disorders, somatic symptoms, related disorders, or depression without suicidal ideation will not be excluded. The screening for this part will be conducted using the Mini International Neuropsychiatric Interview (M.I.N.I.) [26], a widely used structured diagnostic interview that screens for various mental diseases.
4. Antisocial personality disorders
5. Significant suicidal ideation at screening
6. Past or present psychiatric or physical disease that is likely to interfere with continuation and evaluation of the study
7. Any other type of marked chronic pain
8. Taking narcotic analgesics
9. Difficulty in attending 10 sessions as an outpatient during the 16-week CBT implementation period
10. Previously received structured individual CBT
11. Unable to understand verbal and written communication in the Japanese language
12. Pregnant or lactating women
13. Any other person whom the principal investigator has determined to be unsuitable as a participant of the study

Warning symptoms list:

- Symptoms that first appeared after 50 years of age
- Any rectal bleeding that has not undergone sufficient medical investigation (excluding that caused by known hemorrhoids)
- Diarrhea-predominant IBS in which no colonoscopy investigation has been conducted
- Unexplained weight loss without a change in eating habits
- Nocturnal symptoms sufficient to cause insomnia
- The presence of warning findings (anemia, positive inflammatory reactions, or positive fecal occult blood)
- Persons with a family history of colon cancer in a first- or second-degree relative (grandparents, parents, siblings, or children)

#### Participant withdrawal of consent

Participants can withdraw at any time without penalty. Withdrawal information will be collected.

### Randomization

Randomization will be conducted using the Electronic Data Capture System (HOPE eACReSS, Fujitsu ltd.), which can be accessed from each institute via the Internet. The Electronic Data Capture System will be set up and managed independent of the study by the Data Management division at the National Center of Neurology and Psychiatry (NCNP). A random sequence will be generated using stratified randomization by hospital. Eligible participants will be registered at each hospital on a PC terminal connected to the Electronic Data Capture System via the Internet. Allocation to the treatment or control group will be implemented centrally and automatically upon registration and then displayed on the terminal.

### Planned interventions

#### CBT - interoceptive exposure with complementary video materials (CBT-IE-w/vid)

Participants receive CBT using interoceptive exposure for IBS with complementary psychoeducational video materials (CBT-IE-w/vid**)**. The original IE program [15] consists of 10 weekly sessions of approximately 60 min. Our CBT-IE-w/vid intervention consists of 10 weekly sessions, but the time length of each session has been changed. Each session consists of 2 parts: viewing 3- to 13-min videos at home in advance followed by about 30-min of face-to-face therapy session with a therapist at hospital. All CBT-IE-w/vid therapists will be medical doctors who specialize in psychosomatic medicine or psychologists qualified in least one of the following: licensed by the Japanese government, clinical psychologists qualified by the Foundation of the Japanese Certification Board for Clinical Psychologists, or medical psychologists qualified by the Japanese Society of Psychosomatic Medicine.

CBT-IE-w/vid intervention includes (a) education that IBS symptoms reflect conditioned reactions to reminders of gastrointestinal distress (e.g., food intake or a ride on a train); (b) self-monitoring of IBS symptoms; (c) attentional control skills to learn to shift attention away from rather than perseverate upon unpleasant visceral sensations [27]; (d) cognitive therapy to identify threat-laden appraisals of disturbance caused by IBS symptoms; (e) interoceptive exposure involving repeated exposure to visceral sensations (e.g., tightening stomach to produce gut sensations, delaying entrance to the bathroom, eating feared/avoided foods) to reduce fear of the sensations; and (f) in vivo exposure to feared/avoided situations in which IBS sensations were expected (e.g., riding a long-distance train, eating at restaurants, going places where bathrooms are not accessible) while weaning “safety signals” or “safety behaviors” (e.g., keeping medicines handy at all times or carrying additional underclothing). Reliance on safety signals or safety behavior during exposure practices interferes with relearning. Homework, using a paper-based textbook, is required for each session. Table 1 shows the contents of each session, the handout (including homework), and the duration of each video. There is no objective control over the usage of the complimentary video materials. The videos continue to be available after the end therapy, as are texts and materials. Patient usage of video material and homework will be tracked using a compliance check sheet during intervention and at follow up.

**Table 1:** Contents of each session

Session number	Contents of intervention	Handouts	Play time of Video
1	Education about IBS and psychological stress on digestive functioning, awareness-raising	• Personal IBS profile (in session use)	(12′ 16″)
		• Monitoring IBS distress	
2	Education about the role of conditioning in IBS, attentional training	• Monitoring IBS distress	(6′ 44″)
		• Guide for Attentional training	
		• Common IBS symptom appraisal list	
3	Attentional training, cognitive restructuring for IBS sensations and risk estimates	• Monitoring IBS distress	(9′ 31″)
		• Common IBS symptom appraisal list	
4	Cognitive restructuring for symptoms of IBS, valence estimates, hierarchy construction for IBS sensation reminders	• Monitoring IBS distress	(9′ 05″)
		• Deliberate exposure hierarchy	
5	Cognitive restructuring, interoceptive exposure assessment, in vivo exposure	• Monitoring IBS distress	(11′ 36″)
		• Interoceptive exposure exercises	
		• Interoceptive exposure FAQ	
		• Guide for IBS and in-vivo exposure	
		• In-vivo exposure instructions	
		• Deliberate exposure record	
6–9	Conduct of Interoceptive exposure, in vivo exposure	• Monitoring IBS distress	(5′ 40″)
		• Interoceptive exposure instructions	(3′ 32″)
		• Interoceptive exposure record	(3′ 43″) (3′ 35″)
10	Interoceptive exposure, in vivo exposure, summary of the all sessions, relapse prevention	• Monitoring IBS distress	(7′ 44″)
		• Relapse prevention Map	
		• Dealing with setbacks	
		• List of positive Accomplishments	

#### Treatment as usual (TAU)

Patients in both treatment arms will receive TAU by medical doctors. TAU is defined as standard treatment for IBS as recommended by the clinical practice guidelines of the Japanese Society of Gastroenterology [14]. According to the guidelines, a three-step strategy is adopted. The first step consists of lifestyle modification and gut-targeted pharmacotherapy for approximately 4 weeks. Non-responders to the first step intervention proceed to the second step, which includes psychopharmacological agents and brief psychotherapy in addition to the first step interventions for approximately 4 weeks. In the third step, for patients who have not obtained sufficient improvement by the second step, specific psychotherapies including CBT are recommended.

In this study, TAU will consist of only the first and second step interventions. Time spent in each TAU session is 15–30 min. The frequency of TAU sessions is usually once every 4 weeks, but it may vary depending on the patient’s schedule. There is no limit for duration of TAU in either arm. This procedure will be beneficial and ethically desirable for patients.

The medical doctor in charge will implement TAU using the common guidance reviewed by IBS experts (SF, KY, and Hiroe Kikuchi).

#### Quality assurance

To ensure treatment fidelity, CBT-IE-w/vid therapists will have completed designated CBT-IE-w/vid training and will receive continuous support from supervisors (TA, YF) and peer-supervisors (MF, H Kawanishi) throughout the study. All CBT-IE-w/vid sessions will be subject to evaluations of treatment adherence and competence using treatment manuals that provide detailed session-by-session guidance to standardize intervention among all therapists, who will complete checklists for session protocols after each session. Therapists will rate patient adherence to homework using a six-point scale [10].

### Discontinuation

Any participant who meets any of the following discontinuation criteria will have their intervention stopped. When possible, subsequent assessment will be conducted.

1. Participant requests discontinuation or participant withdraws consent
2. Difficulty continuing the intervention because of a serious adverse event
3. Participant proves after assignment not to fulfill the eligibility criteria
4. Participant with suicide risk
5. Any other reason that the primary investigator, the medical doctor, CBT-IE-w/vid therapist, supervisor, or the Data and Safety Monitoring Board agree warrants discontinuation
6. The entire clinical trial is stopped

## Measures

### Primary outcome measure

1. ***Japanese version of the IBS severity index (IBS-SI-J)***: IBS-SI or IBS severity scoring system (IBS-SSS) was developed in the UK and is widely used to assess the severity of lower gastrointestinal symptoms and the degree to which the quality of life is impaired by IBS. IBS-SI-J is a valid, reliable, and appropriate instrument for detecting and assessing the severity of IBS status in Japanese patients [24, 25]. This self-report instrument has five items that score abdominal pain, abdominal distention, bowel movements, and quality of life. The total score ranges from 0 to 500. IBS severity is graded as mild (75–174), moderate (175–299), or severe (300–500).

### Secondary outcome measures

1. ***Visceral Sensitivity Index (VSI)***. The VSI assesses gastrointestinal symptom-specific anxiety [28, 29]. It consists of 15-items scored with a six-point Likert scale, with items such as “When I feel discomfort in my belly, it frightens me.” Lower scores indicate more severe anxiety for abdominal symptoms.
2. ***Japanese version of the Irritable Bowel Syndrome Quality of Life Scale (IBS-QOL-J).*** The IBS-QOL-J assesses disease-specific QOL for IBS [30]. It consists of 34 items scored with a five-point Likert scale. Items ask how IBS affects the daily functioning of the participant. It includes eight subscales: dysphoria, interference with activity, body image, health worry, food avoidance, social reaction, sexual concerns, and relationships.
3. ***IBS Diary modified for weekly use.*** The IBS Diary [31], which was modified for weekly use, records stool form types and frequencies, abdominal symptom severity scores (e.g., pain/discomfort rated on a 10-point ordinate scale), subjective feelings of stress, medication adherence, and the number of times “medicines to be taken as needed” were used in a week.
4. ***Irritable Bowel Syndrome Global Improvement Scale (GIS)***. The GIS assesses participants’ subjective improvement [32] scored on a seven-point Likert scale that ranges from 1 (substantially improved) to 7 (substantially worse).
5. ***MOS 36-Item Short-Form Health Survey (SF-36).*** The SF-36 assesses health related QOL and consists of 36 items [33] that measure eight health concepts: physical functioning, physical role, bodily pain, general health, vitality, social functioning, emotional role, and mental health.
6. ***Beck Depression Inventory-II (BDI-II).*** The BDI-II assesses the existence and severity of depression symptoms, such as sadness and suicidal ideation [34, 35]. It consists of 21-items scored on a four-point Likert scale.
7. ***State-Trait Anxiety Inventory (STAI)***. The STAI assesses state and trait anxiety [36, 37] and consists of 40-items scored on a four-point Likert scale.
8. ***Japanese Version of the Body Vigilance Scale (BVS*-J)**. The BVS-J assesses attention to bodily sensations [38]. It consists of four items, three of which assess the degree of attentional focus, perceived sensitivity to changes in bodily sensations, and the average amount of time spent attending to bodily sensations. The fourth item involves separate ratings for attention to 15 bodily sensations (e.g., heart palpitations, abdominal discomfort).
9. ***Cognitive Control Scale.*** The Cognitive Control Scale assesses the degree of cognitive control [39] and consists of 12 items, scored on a four-point Likert scale, that ask about thoughts that occur during a disturbing event. This scale has a two-factor structure, “analysis of thought and behavior” and “reframing.”
10. ***Medication changes***. Medication changes include an additional or increased dose of any drug other than sleeping drugs and “medicines to be taken as needed” in response to a participant request or worsening disease. Medication change data will be recorded from the start of intervention.

Table 2 shows the schedule of assessments of outcomes.

**Table 2:**
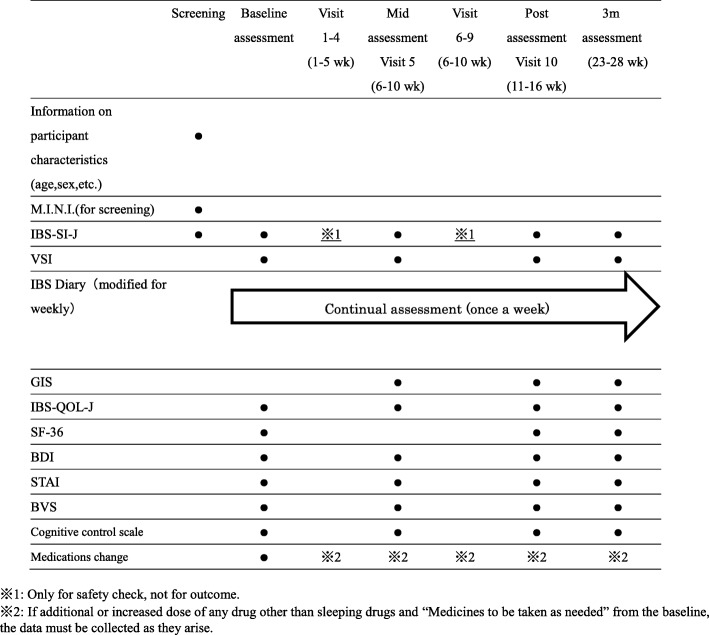
Schedule of assessments

### Sample size

We estimated a sample size of 60 per group (total 120) from standardized treatment effects that were estimated as 0.91 to 1.02 based on the single-arm feasibility study that preceded this study [21]. However, we used a conservative estimate of 0.6 for the standardized treatment effects for between-group differences because the previous randomized control trial [15] with a similar target population and outcomes estimated standardized treatment effects at 0.33 to 0.70. Thus, the minimum required sample size with minimum power set at 0.8 was estimated as 45 subjects per group. We increased this to 60 subjects per group to maintain adequate statistical power in case of potential dropouts.

### Statistical analysis

Statistical analyses will be conducted by an independent statistician (KM) who is not a TAU medical doctor or CBT-IE-w/vid therapist.

- Analysis set for efficacy analysis: modified intention to treat (ITT) set that includes all subjects who have at least one outcome after intervention.
- If possible, data from participants who drop out will be collected after dropout.
- Analysis for background factors: summary statistics (frequency, mean, SD) will be calculated depending on the characteristics of each variable.
- Primary analysis: mixed model for repeated measures (MMRM) analysis will be applied to the change from baseline for IBSSI-J at visit five (for participants in the TAU alone group the time point is defined as 6 to 10 weeks after randomization) and visit ten (for participants in the TAU alone group the time point is defined as 10 to 16 weeks after randomization), where treatment, visit, treatment-by-visit interaction, and baseline IBSSI-J are modeled as fixed effects. T-tests will then be conducted on the difference of adjusted means between the groups at visit 10.
- Secondary analyses: MMRM analysis will be applied to other continuous outcomes.

### Data management and monitoring

Acquired data will be entered immediately into an Electronic Data Capture System at each center. Data review and verification will be conducted by a person who did not perform the initial entry. Onsite monitoring will be conducted periodically by dedicated staff of the Data Management division at NCNP (these staff members are independent from this study). The Data and Safety Monitoring Board will oversee the trial data and ethics, with an independent chair and two independent members. The members of the committee are independent from the sponsor and competing interests of the present study. The principle investigator (TA) will submit an annual progress report to the Data and Safety Monitoring Board.

### Safety

Information on serious adverse events (SAEs) will be collected from the TAU medical doctor or CBT-IE therapist during the intervention. Based on the Ethics Guideline for Clinical Research (Ministry of Health, Labour and Welfare (MHLW)), an SAE is defined as ‘an adverse event that may lead to death or to enduring severe impairment depending on the patient’s conditions and circumstances’ and will include any one of the following outcomes:

- Results in death (all deaths regardless of causal relationship with the intervention or for which a causal relationship with the intervention cannot be excluded, during the intervention phase or up to 30 days after the completion of the intervention)
- Is life threatening, or places the participant at immediate risk of death from the event as it occurred
- Requires or prolongs hospitalization
- Causes persistent or significant disability or incapacity
- Results in congenital anomalies or birth defects
- Is another condition which investigators judge to represent significant hazards

### Follow-up after serious adverse event

When an SAE occurs, the medical doctor will take all necessary and appropriate measures to ensure the safety of the patient and will provide appropriate treatment including hospital admission. The medical doctor will immediately notify the principal investigator (TA) of the SAE, and the principal investigator will notify all collaborating investigators. The head investigator of all sites will report the SAE to the relevant authorities (Data and Safety Monitoring Board, Institutional Review Board, and President of each center) and the Japanese MHLW. The medical expenses will be borne by the patient because the treatment will be supplied as a healthcare service provided under national health insurance, the same as for usual treatment. There will be no special financial compensation; however, any negligence on the part of the physician may be covered by the doctors’ liability insurance.

### Ethical issues

The present study complies with the ethical guidelines for clinical studies published by the Japanese MHLW, as well as the ethical principles established for research on humans stipulated in the Declaration of Helsinki and further amendments thereto. This protocol has been reviewed and approved by the Institutional Review Board of the (Japanese) National Center of Neurology and Psychiatry (accepted on November 29, 2017; A2017–067) and the Ethics Committees of all four collaborating facilities.

If important protocol modifications such as changes to eligibility criteria, outcomes, or analyses are needed for any reason, the principal investigator (TA) will communicate this with the Institutional Review Boards.

Written informed consent will be obtained from all participants, who will be informed that they can withdraw from the study at any time and that their withdrawal will never lead to refusal of any other services. A medical doctor or a clinical psychotherapist in charge of the informed consent process is responsible for ensuring that informed consent is given by each patient.

Data from each participant will be handled with sequentially allocated numbers to maintain participant confidentiality. All data collected in this study will be securely stored without personal information (name, address, etc.). Access to the data is encrypted and limited to researchers affiliated with this study.

### Dissemination

The study findings will be disseminated via publications in peer-reviewed international journals. We will submit annual progress reports to the MHLW and NCNP, which are funding resources for this trial. We will also present the findings at relevant research conferences.

## Discussion

Previous studies have indicated that the standard medications (TAU), such as bulking agents and antispasmodics, have low responsiveness [40–42] and that many patients with IBS suffer from ongoing symptoms. Some meta-analyses on the efficacy of antidepressants for IBS reported that they are effective in IBS treatment [43], but several systematic reviews [44] have indicated that the efficacy of SSRI and tricyclic antidepressants did not surpass bulking agents and antispasmodics. In such circumstances, psychological treatments such as CBT have been shown to be helpful for IBS symptoms [45, 46]. However, Craske et al. [15] reported that the use of an active control leads to smaller effect sizes than studies with TAU or wait-list designs. Therefore, in this study we will assess the effectiveness of CBT-IE-w/vid in addition to TAU compared to TAU alone for improving IBS symptoms.

CBT-IE was originally developed for panic disorder and involves exercises causing physical sensations that mimic those experienced in a panic attack [16, 47]. Patients with IBS also have problems with physical sensations such as hypervigilance and hypersensitivity to sensations related to IBS symptoms [15]. Although the interoceptive exposure procedure can be a promising approach to reduce anxiety to abdominal sensation as a perpetuating factor of IBS, it has never been studied in the Japanese population. As a result, this procedure is not used clinically for IBS in Japan. In this study, we will evaluate gastrointestinal symptom-specific anxiety using VSI and body vigilance with BVS-J as possible mediators on the effect of CBT-IE on IBS symptoms. We will also assess IBS specific QOL and health related QOL as secondary outcomes. Efficacy on QOL has not been well elucidated in the previous CBT-IE study by Craske et al.

Our study has three strengths. First, it is the first RCT of CBT treatment for IBS in Japan. Second, we will utilize video materials to facilitate and shorten each face-to-face therapy session to enhance the cost-effectiveness of psychotherapy. The amount of the time necessary for a face-to-face therapy session was reduced from 66 min (face-to-face therapy alone) [21] to 38 min (using complementary video materials) [22], and the program relies more on self-administered management. Third, this is a multisite RCT (five sites in Japan), which will facilitate participant recruitment. In addition, participants recruited from five sites will provide a more reliable representation of the target patient population than a single-center study, which is favorable to implementation as well.

There are some limitations to this study. The primary endpoint in this study is a patient-reported outcome (PRO). It is desirable to set more objective outcomes as primary endpoints. However, currently there is no standard objective outcome for IBS that could be used as an objective primary endpoint. Using PRO as the primary endpoint is recommended in the guidance for clinical trials of IBS published by the United States Food and Drug Administration [48]. Moreover, bias from using PRO is expected to operate equally in both groups, rendering any observed between-group differences clinically meaningful. Also, this study is a non-blinded randomized controlled trial due to the type of intervention (psychotherapy). The impossibility of effectively blinding participants to treatment allocation in a CBT study also makes it impossible to control for placebo effects. This is a general problem in all psychotherapy studies [49].

## Abbreviations

CBT: Cognitive behavioral therapy
CBT-IE: Cognitive behavioral therapy using interoceptive exposure
CBT-IE-w/vid: CBT-IE with complimentary video materials
IBS: Irritable bowel syndrome
ITT: Intention to treat
PRO: patient-reported outcome
QOL: Quality of life
RCT: Randomized controlled trial
SAE: Serious adverse event
TAU: Treatment as usual
